# Detection of breast cancer cells using targeted magnetic nanoparticles and ultra-sensitive magnetic field sensors

**DOI:** 10.1186/bcr3050

**Published:** 2011-11-03

**Authors:** Helen J Hathaway, Kimberly S Butler, Natalie L Adolphi, Debbie M Lovato, Robert Belfon, Danielle Fegan, Todd C Monson, Jason E Trujillo, Trace E Tessier, Howard C Bryant, Dale L Huber, Richard S Larson, Edward R Flynn

**Affiliations:** 1 Department of Cell Biology & Physiology, University of New Mexico School of Medicine, MSC08 4750, 1 University of New Mexico, Albuquerque, NM 87131, USA; 2 Cancer Research & Treatment Center, University of New Mexico School of Medicine, MSC07 4025, 1 University of New Mexico, Albuquerque, NM 87131, USA; 3 Department of Pathology, University of New Mexico School of Medicine, MSC08 46401 University of New Mexico, Albuquerque, NM 87131, USA; 4 Department of Biochemistry & Molecular Biology, University of New Mexico School of Medicine, MSC08 4670, 1 University of New Mexico, Albuquerque, NM 87131, USA; 5 Senior Scientific LLC, 800 Bradbury SE, Albuquerque, NM 87106, USA; 6 Nanomaterials Sciences Department, Sandia National Laboratories, PO Box 5800, Albuquerque, NM 87185, USA; 7 Center for Integrated Nanotechnologies, Sandia National Laboratories, PO Box 5800, Albuquerque, NM 87185, USA

## Abstract

**Introduction:**

Breast cancer detection using mammography has improved clinical outcomes for many women, because mammography can detect very small (5 mm) tumors early in the course of the disease. However, mammography fails to detect 10 - 25% of tumors, and the results do not distinguish benign and malignant tumors. Reducing the false positive rate, even by a modest 10%, while improving the sensitivity, will lead to improved screening, and is a desirable and attainable goal. The emerging application of magnetic relaxometry, in particular using superconducting quantum interference device (SQUID) sensors, is fast and potentially more specific than mammography because it is designed to detect tumor-targeted iron oxide magnetic nanoparticles. Furthermore, magnetic relaxometry is theoretically more specific than MRI detection, because only target-bound nanoparticles are detected. Our group is developing antibody-conjugated magnetic nanoparticles targeted to breast cancer cells that can be detected using magnetic relaxometry.

**Methods:**

To accomplish this, we identified a series of breast cancer cell lines expressing varying levels of the plasma membrane-expressed human epidermal growth factor-like receptor 2 (Her2) by flow cytometry. Anti-Her2 antibody was then conjugated to superparamagnetic iron oxide nanoparticles using the carbodiimide method. Labeled nanoparticles were incubated with breast cancer cell lines and visualized by confocal microscopy, Prussian blue histochemistry, and magnetic relaxometry.

**Results:**

We demonstrated a time- and antigen concentration-dependent increase in the number of antibody-conjugated nanoparticles bound to cells. Next, anti Her2-conjugated nanoparticles injected into highly Her2-expressing tumor xenograft explants yielded a significantly higher SQUID relaxometry signal relative to unconjugated nanoparticles. Finally, labeled cells introduced into breast phantoms were measured by magnetic relaxometry, and as few as 1 million labeled cells were detected at a distance of 4.5 cm using our early prototype system.

**Conclusions:**

These results suggest that the antibody-conjugated magnetic nanoparticles are promising reagents to apply to in vivo breast tumor cell detection, and that SQUID-detected magnetic relaxometry is a viable, rapid, and highly sensitive method for in vitro nanoparticle development and eventual in vivo tumor detection.

## Introduction

New cases of invasive breast cancer were predicted to exceed 207,000 in the US, where an estimated 39,840 women died of breast cancer in 2010 [1]. Currently, detection is routinely done by mammogram, which has significantly improved breast cancer outcomes, but mammograms cannot distinguish between benign and malignant lesions [2]; biopsy is required to confirm or rule out cancer. Furthermore, tumors in dense or scarred breast tissue or in augmented breasts are difficult to detect by mammography, and the best estimates suggest that mammography fails to detect 10% to 25% of breast cancers [3]. Improvements in breast cancer detection, particularly with technology that can distinguish malignant from benign lesions, improve upon the current sensitivity and, if applied to radio-opaque breasts, would be a tremendous advance. In addition, the ideal technology will be inexpensive and rapid and can be accomplished with little or no discomfort to the patient.

Increasing specificity in breast cancer detection will require the use of specific markers that can distinguish between malignant and benign lesions. The ideal marker would have high specificity toward cancer cells relative to normal cells and the target(s) would be represented on a high proportion of tumor types. Although great progress has been made in this field and many promising targets have been identified, the ideal target remains elusive [4]. An alternative strategy involves the use of marker cocktails, allowing the development of unique combinations for individual patients with different tumor expression profiles. This is most feasible in follow-up and therapeutic settings since the cancer has already been identified and characterized. In anticipation of the identification of new markers in the future and the possibility of using cocktails, we are focusing on the development of a universal probe, based on iron oxide nanoparticles, and developing a universal conjugation method to allow targeting by any antibody or peptide to tumor cell surface targets. This strategy will allow the probe to be targeted to new molecules as they are discovered and allow the development of personalized cocktails based on individual patient histology. In the development phase, described here, we have selected human epidermal growth factor-like receptor 2 (Her2), a surface antigen that is overexpressed in approximately 30% of breast cancers [5]. Her2 is well characterized, and a variety of antibody-based targeting methods are available; therefore, Her2 is an ideal prototypical breast cancer cell surface target.

The use of magnetic nanoparticles conjugated to tumor-specific probes combined with detection of these particles through measurement of their relaxing fields following a magnetization pulse represents a promising new technology that has the potential to improve our ability to detect tumors earlier because of high theoretical sensitivity [6]. We have developed a novel nanotechnology method based on the use of magnetic nanoparticles labeled with specific antibodies for breast cancer and ultra-sensitive detection of these particles by using Superconducting Quantum Interference Device (SQUID) sensors [7]. Magnetic relaxometry [6,8-10] for detection of targeted magnetic nanoparticles is fast and theoretically is more specific than magnetic resonance imaging detection since only particles bound to their targets are detected, eliminating the problems associated with signals from unbound particles. The magnetic moments observed by magnetic relaxometry are also linear with the number of nanoparticles bound to the tumor and may be used to determine the number of cancer cells in the tumor [8]. In magnetic relaxometry, magnetic nanoparticles that have been conjugated to antibodies or other agents are incubated with live cells [11]. After a brief period, the nanoparticles attach to the targeted cells in large numbers, typically on the order of 100,000 nanoparticles per cell [11,12]. A magnetizing pulse of less than 1 second is applied with a set of Helmholtz coils to achieve a uniform magnetizing field over the sample. A field of 40 gauss is adequate to appreciably polarize these nanoparticles, which are typically 25 nm in diameter, resulting in an induced collective magnetic moment. After the magnetizing field is removed, the magnetic moment decays through the Néel mechanism [13] with a time constant on the order of 1 second. This decaying field is measured by an array of second-order gradiometer SQUID sensors [6].

Our long-term goal is to develop magnetic nanoparticle-based magnetic imaging to detect *in vivo *malignancies with high sensitivity and specificity. To this end, we first set out (a) to identify appropriate magnetic nanoparticles for SQUID-detected magnetic relaxometry of breast cancer, (b) to develop reproducible methods for conjugating antibody or peptide probes, (c) to identify appropriate *in vitro *breast cancer models with which to test conjugated nanoparticle binding specificity, and (d) to determine the ability of magnetic relaxometry to specifically detect conjugated nanoparticles bound to cells. We demonstrate that we have achieved anti-Her2 antibody conjugation to magnetic nanoparticles and that these antibody-conjugated nanoparticles display magnetic properties that make them ideal for magnetic detection with SQUID sensors. Furthermore, antibody-conjugated nanoparticles bind in greater numbers to breast cancer cells expressing high levels of Her2 compared with cells expressing low Her2. Our results suggest that this approach is a promising first step toward the development of novel magnetic-based cancer detection *in vivo*.

## Materials and methods

### Materials

Carboxyl-functionalized iron oxide nanoparticles (SHP-30 Lot SAO5) with a nominal diameter of 30 nm were purchased from Ocean NanoTech (Springdale, AR, USA). N-hydroxysulfosuccinimide (Sulfo-NHS) and 1-ethyl-3-[3-dimethylaminopropyl]carbodiimide hydrochloride (EDC) were purchased from Pierce (Rockford, IL, USA). Trypan blue, albumin solution from bovine serum, sodium azide, potassium ferrocyanide, NaHCO_3_, and ethylenediaminetetraacetic acid (EDTA) were purchased from Sigma-Aldrich (St. Louis, MO, USA). Anti-Her2 antibody was purchased from Bender MedSystems (now part of eBioscience, Inc., San Diego, CA, USA). Phosphate-buffered saline (PBS) was purchased from Gibco-BRL (now part of Invitrogen Corporation, Carlsbad, CA, USA), and fetal bovine serum (FBS) was purchased from HyClone (Logan, UT, USA). Diff-Quik stain was purchased from Dade Behring (now part of Siemens, Munich, Germany) and Brazilliant stain was purchased from Anatech Ltd. (Battle Creek, MI, USA). Xanthene dye was purchased from Siemens. Cytoseal XYL was purchased from Richard-Allan Scientific (now part of Thermo Fisher Scientific Inc., Waltham, MA, USA). Matrigel™ recombinant basement membrane was purchased from Fisher Scientific (Pittsburgh, PA, USA).

### DC susceptometry

DC magnetic characterizations of stock nanoparticles were performed with an MPMS-7 SQUID magnetometer system (Quantum Design, San Diego, CA, USA). DC magnetization curves were acquired by equilibrating the sample at the measurement temperature in zero field, incrementally increasing the field, and pausing 100 seconds at each field before measurement. Five sequential measurements were taken at each field, a mean of those measurements was calculated, and the three values with the lowest deviation from the mean were averaged and reported as the moment. Zero-field cooled curves were determined by cooling the sample in the absence of a magnetic field to 5 K and then slowly warming in a 1-mT field. After thermal equilibration at a target temperature, a series of five measurements was taken and the values were processed as described to obtain the magnetization value.

### Transmission electron microscopy

The nanoparticles were imaged by transmission electron microscopy (TEM) by using a Tecnai G^2 ^F30 transmission electron microscope at 300 kV (FEI Corporation, Hillsboro, OR, USA). Size distributions were determined from the TEM images by using ImageJ (public domain software from the National Institutes of Health). Briefly, the Feret diameter (defined as the maximum caliper diameter) was measured from a sample of approximately 1,000 particles selected from multiple TEM images. Particles in contact with the edge of an image were automatically excluded, and overlapping particles were manually excluded from the size analysis.

### Magnetic relaxometry

To measure the desired Néel relaxation of the nanoparticles by relaxometry, the particles must be immobilized. In the case of cell samples, the antibody-conjugated nanoparticles were immobilized by the binding of the antibodies to receptors on the cell surface (described below). For calibration purposes, a known quantity of the same nanoparticle solution was applied to a Q-tips cotton swab (Unilever, Trumbull, CT, USA) and allowed to dry in air.

In this study, the nanoparticles were subjected to a magnetizing field of approximately 40 gauss for 0.75 seconds followed by a delay of 35 milliseconds and subsequent detection every millisecond of the relaxing magnetic field for 2.2 seconds by using a seven-channel SQUID sensor array (BTi 2004; 4D-Neuroimaging, San Diego, CA, USA), which has been described in detail elsewhere [6]. The SQUID sensors operate in a non-shielded environment, achievable by the second-order gradiometer cancellation of background fields, and have a noise floor of approximately 2 pT/√Hz. This sequence was repeated 10 times and the result was signal-averaged to increase the signal-to-noise ratio. The samples were uniformly polarized (parallel to the center gradiometer axis) by using a 49-cm diameter, 100-turn Helmholtz pair powered by a 5-kW current-regulated supply (Sorenson SGA 80/63). The current through the magnetizing coils was monitored by a Hall effect transducer to ensure constant magnetic fields. The relaxometry data were recorded with a National Instruments PXI8336 16-channel digitizer (National Instruments Corporation, Austin, TX, USA).

Data analysis was performed with the Multi-Source Analysis program, written in our lab by using MATLAB (The MathWorks Inc., Natick, MA, USA). After removal of 60-cycle contamination and other artifacts that occur because of external noise, the data were signal-averaged. Background data (acquired with no sample) were subtracted from the sample data to compensate for background fields arising from induced currents following the magnetic field pulse. The relaxation curves were fit by a logarithmic function at long times (to determine the DC offset) and an exponential function at short times to obtain the magnetic field amplitude at each sensor position [6]. To solve the electromagnetic inverse problem, we fit the spatial dependence of the magnetic field by modeling the sample as one or more discrete magnetic dipoles, which allows us to determine the location (x, y, z) and magnetic moment (m_z_) of each dipole. In solving the inverse problem by this modeling approach, we used the fact that the direction of the magnetic dipoles induced in the source is parallel to the applied magnetizing field. Knowledge of the vector direction of the magnetic dipole moments yielded increased precision in determining the spatial coordinates of the sources. The least-squares fit was performed by using the Levenberg-Marquardt algorithm. To determine the spatial coordinates and moments for multiple discrete sources, data were obtained by using n different sample positions - equivalent to a sensor array with 7n elements - such that 7n (the number of field amplitudes obtained) exceeds 4s (the number of unknowns for s discrete sources). Confidence limits (95%) obtained from fitting the data to a dipole model indicate that 1-mm accuracy was typically obtained for a magnetic moment on the order of 10^-7 ^J/T or greater located several centimeters from the sensor system.

### Iron assay

The iron concentration (mg[Fe] per milliliter) of nanoparticle samples was determined destructively by dissolving them in acid, forming the phenanthroline/Fe^2+ ^complex, and then quantifying the concentration of a known dilution spectrophotometrically [14].

### Cell lines and flow cytometry

MCF7, BT-474, and MDA-MB-231 breast cancer cells and Chinese hamster ovary (CHO) cells were purchased from the American Type Culture Collection (Manassas, VA, USA). MCF7 cells transfected to overexpress Her2 antigen, designated MCF7/Her2-18, were kindly provided by Mien-Chie Hung (The University of Texas M. D. Anderson Cancer Center, Houston, TX, USA) [15]. MCF7/Her2-18 cells were cultured in advanced Dulbecco's modified Eagle's medium/F-12 medium supplemented with 10% FBS (vol/vol) 1% penicillin streptomycin (vol/vol) and 4 μg/mL ciprofloxacin. MDA-MB-231 cells were cultured in Leibovitz's L-15 medium supplemented with 10% FBS (vol/vol), 1% penicillin streptomycin (vol/vol), and 4 μg/mL ciprofloxacin. CHO cells were cultured in RPMI 1640 medium supplemented with 10% FBS (vol/vol), 1% penicillin streptomycin (vol/vol), and 4 μg/mL ciprofloxacin. MCF7/Her2-18 and parental CHO cells were cultured in an incubator at 37°C with 5% CO_2 _and maintained at a cell concentration of between 1 × 10^5 ^and 1 × 10^6 ^viable cells/mL. MDA-MB-231 cells were cultured in an incubator at 37°C with no CO_2 _and maintained at a cell concentration of between 1 × 10^5 ^and 1 × 10^6 ^viable cells/mL. For antibody and nanoparticle labeling of attached cells, cells were cultured on acid-washed glass coverslips. Quantification of Her2 site density was performed as previously described by using approximately 10^5 ^cells and an anti-human p185^Her2 ^(cell-erythroblastic leukemia viral oncogene homolog 2, or c-*erb*B2) fluorescein isothiocyanate (FITC) antibody (Invitrogen Corporation) [11].

### Nanoparticle conjugation

The antibody was attached to the nanoparticles by using the carbodiimide method, as previously described [11], with the exception of centrifugation speed, which was increased to 7,500 *g *to account for the use of smaller (30 nm) nanoparticles. Anti-Her2-conjugated nanoparticles were stored at 4°C prior to use.

### Cell labeling and sampling

MCF7/Her2-18, parental CHO, or MDA-MB-231 cells were harvested with EDTA and washed with sterile PBS. Harvested cells were counted by using 0.4% Trypan blue solution on a hemocytometer (Hausser Scientific, Horsham, PA, USA). Each sample consisted of 7.5 × 10^6 ^cells suspended in 200 μL of cold media to which 0.8 mg of anti-Her2/neu-coupled nanoparticles was added. Samples in 1.5-mL microcentrifuge tubes were centered under the seven-channel SQUID sensor array. Cells and Her2/neu-nanoparticles were incubated on ice for 15 minutes, and SQUID measurements were taken every 2 minutes, starting at 1 minute after nanoparticle addition.

Cytospin slides were prepared by adding 200 μL of bovine serum albumin solution with 5 μL of cell/nasopharyngeal sample to a cytofunnel. The slides were then placed in a Shandon Cytospin 4 machine (Thermo Fisher Scientific Inc.) and centrifuged at 1,100 *g *for 7 minutes. Slides were stained with either Diff-Quikstain, which is similar to a Wright-Giemsa stain, or Prussian blue stain, which reveals the presence of iron. For Prussian blue stain, slides were then fixed by dipping five times in 0.01% sodium azide in 1 g/L xanthene dye. Potassium ferrocyanide solution (a 1:1 solution of 20% of hydrochloric acid and 10% potassium ferrocyanide) was prepared fresh, applied directly to the cell sample on the slide, and incubated in the dark for 20 minutes. The slides were then dipped in double-distilled water three times. Brazilliant was applied directly to the cell sample on the slide and incubated in the dark for 5 minutes. After Prussian blue or Diff-Quik staining, the slides were dipped in double-distilled water three times and allowed to dry. The slides were then cover-slipped with Cytoseal XYL. Stained samples were qualitatively assessed for nanoparticle attachment by using light microscopy. Light microscopy was performed on an Axiovert 200 MAT microscope (Zeiss, Munich, Germany), and images were captured with a Moticam 2300 camera and Motic Images Plus software (Motic, Xiamen, China).

### Confocal microscopy

Cells grown on glass coverslips were incubated with nanoparticles as described above and then fixed in 4% paraformaldehyde in PBS. After several washes in PBS, cells were blocked in PBS containing 5% normal goat serum (NGS) and then incubated in goat anti-mouse IgG conjugated to Alexa 488 (Invitrogen Corporation) (diluted 1/250 in PBS/NGS) for 45 minutes at room temperature. During the last 20 minutes of incubation, 26 nM rhodamine-conjugated phalloidin (Invitrogen Corporation) was added in order to label actin filaments. After washing in PBS, cells were incubated in 4',6-diamidino-2-phenylindole (DAPI) or Topro-3 (Invitrogen Corporation) to counterstain nuclei and were inverted onto a drop of anti-fade mounting media on a glass slide. Images were captured on a Zeiss 510 confocal microscope and were further manipulated (channels merged and labels added) by using Adobe Photoshop software (Adobe Systems, Inc., San Jose, CA, USA).

For immunofluorescence assays, cells were fixed in 4% paraformaldehyde, washed, and then incubated overnight at 4°C in anti-Her2 antibody (clone 2G11; Bender MedSystems) diluted 1/300 in PBS/NGS. After washing in PBS, cells were incubated in goat anti-mouse IgG-Alexa 488 and rhodamine phalloidin, washed, and mounted as described above.

### Generation of xenograft tumors in mice

B6.129S7-*Rag1^tm1Mom^*/J mice were purchased from The Jackson Laboratory (Bar Harbor, ME, USA), and athymic nude mice were purchased from Harlan Laboratories (Indianapolis, IN, USA). Two to seven days prior to injection of cells, mice were implanted with a 17β-estradiol pellet (1.7 mg, 60-day release; Innovative Research of America, Sarasota, FL, USA). MCF7/Her2-18 cells (1.5 × 10^6^) were injected with 0.150 mL of Matrigel™ into each hind limb flank. Tumor growth was followed by using calipers, and all mice were used when tumors reached around 1 cm by 1 cm. Mice were killed by cervical dislocation under isofluorane anesthesia. Tumors were excised, cut into slices, and injected with 0.175 mg of either anti-Her2 antibody-conjugated or unconjugated nanoparticles by using multiple small injections (total volume of 17.5 μL). Tumor slices were incubated with nanoparticles for 15 minutes and washed in PBS with agitation to remove unbound nanoparticles, and then bound nanoparticles were detected by SQUID relaxometry. Tumor slices were then fixed in 4% paraformaldehyde and processed for Prussian blue staining of paraffin-embedded sections (TriCore Laboratories, Albuquerque, NM, USA). All animal procedures were performed in accordance with the National Institutes of Health *Guide for the Care and Use of Laboratory Animals *and approved by the Institutional Animal Care and Use Committee.

## Results and discussion

### Characterization of nanoparticles demonstrated a narrow size distribution and strong magnetic moment

**Figure 1:**
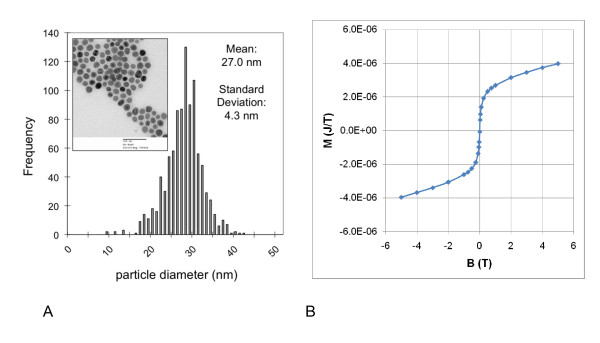
**Characterization of SAO5 nanoparticles**. **(a) **SAO5 nanoparticles were imaged by transmission electron microscopy (inset). The diameters of approximately 1,000 nanoparticles were measured, and the mean and standard deviation were determined. **(b) **SAO5 nanoparticles dried onto a cotton tip were measured by DC susceptometry to determine the total magnetic moment (M) as a function of applied field (B).

Several lots of iron oxide magnetic nanoparticles were evaluated by magnetic relaxometry to determine the lot that yielded the maximum detectable magnetic moment per mg[Fe]. For consistency, all subsequent experiments were performed with this lot (SHP-30 lot SAO5). Theoretically, the relaxation time of bound nanoparticles has a strong (exponential) dependence on the particle volume [13] and therefore it is critical that the nanoparticles fall within a narrow diameter range (near 25 nm) to ensure that their relaxation times are detectable on the timescale of the relaxometry measurement (35 to 2,200 ms, in this case). Figure 1a (inset) shows a TEM image of the SAO5 nanoparticles, which are composed of a single magnetic core of relatively uniform size, coated with a thin layer of polymer, and functionalized with carboxyl groups. The size distribution shown in Figure 1a was obtained by analyzing approximately 1,000 nanoparticles from multiple TEM fields and yielded an average particle diameter of 27 nm, which is in close agreement with the nominal size of 30 nm, and a standard deviation of 4.3 nm. The magnetic moment per mg[Fe] detected by relaxometry was 7.3 × 10^-7 ^J/T per mg[Fe], which represents a threefold improvement in detection sensitivity compared with previous studies [11,16] using multi-core particles.

Nanoparticles (10 μL containing 0.049 mg[Fe]) were immobilized on a cotton tip to characterize their magnetic properties by susceptometry. At room temperature, the magnetization curve (Figure 1b, M versus B) shows that the nanoparticle moment is not completely saturated at 6 T. Measurement of the magnetic moment as a function of temperature (not shown) revealed an average blocking temperature of 350 K, indicating that, at room temperature, only some particles are unblocked and contribute to the relaxometry signal. Blocked particles exhibit relaxation times that are too slow for detection by relaxometry. A numerical fit to the magnetization curve (solid line) suggests that, at a higher field, the nanoparticle moment would reach 5.5 × 10^-6 ^J/T, implying that the saturation magnetization (M_s_) is 81 J/T per kg [Fe_3_O_4_], which is slightly lower than the saturation magnetization of bulk magnetite (92 J/T per kg). These data further imply that the magnetic moment observed by relaxometry is slightly less than 1% of the saturation magnetic moment observed by DC susceptometry (Figure 1b), and this is consistent with the observation that many particles are larger than the ideal diameter of 25 nm (Figure 1a) and therefore are blocked at room temperature.

### Breast cancer cell lines express distinct and measurable Her2 expression levels

**Figure 2:**
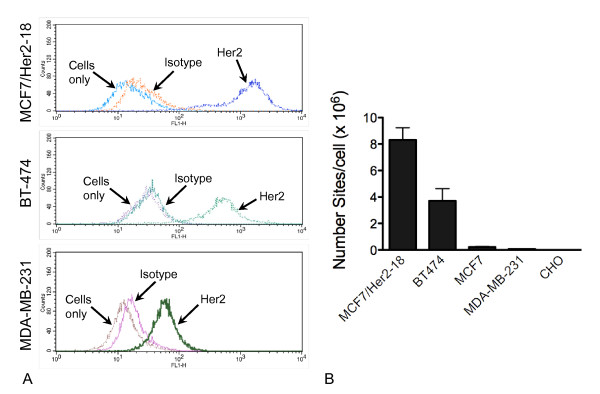
**Characterization of Her2 expression on breast cancer cell lines**. We examined several breast cancer cell lines to identify cells that expressed Her2. The number of Her2-binding sites was determined by flow cytometry with anti-Her2 antibody conjugated to fluorescein. **(a) **Flow cytometric profiles, with cell number plotted as a function of fluorescence intensity, for three representative breast cancer cell lines are shown. **(b) **The number of Her2 sites per cell is calculated by comparison with a range of microspheres with known binding capacities. As expected, MCF7 breast cancer cells engineered to overexpress Her2-18 have a very high number of Her2-binding sites per cell (11.28 × 10^6^), followed by BT-474 breast cancer cells (2.75 × 10^6^), MCF7 breast cancer cells (0.18 × 10^6^), and MDA-MB-231 breast cancer cells (0.11 × 10^6^). The Chinese hamster ovary (non-breast) cell line has a very low number of Her2-binding sites per cell (< 4,000). Her2, human epidermal growth factor-like receptor 2.

Initially, we identified the Her2 tyrosine kinase as an appropriate surface antigen target to assess the efficiency and sensitivity of detection of nanoparticles by using SQUID relaxometry. We quantified Her2 receptor levels on several cell lines reported to express varying levels of Her2, including breast cancer cell lines MCF7/Her2-18 (an MCF7 clone stably transfected with Her2) [15], MCF7, BT-474, and MDA-MB-231 [17] as well as several non-breast cell lines. The number of Her2-binding sites was determined by flow cytometry with anti-Her2 antibodies conjugated to the fluorescent probe FITC. Flow cytometric profiles, with cell number plotted as a function of fluorescence intensity, for three representative breast cancer cell lines are shown in Figure 2a. The number of Her2 sites per cell is calculated as described in Materials and methods, and results are shown in Figure 2b. As expected, MCF7 cells engineered to overexpress Her2-18 have a very high number of Her2-binding sites per cell (8.3 × 10^6^), followed by BT-474 (3.7 × 10^6^), MCF7 (0.23 × 10^6^), and MDA-MB-231 (0.07 × 10^6^). Several non-breast cell lines have a very low number of Her2-binding sites per cell; CHO cells (< 4,000) are shown. Another report describes quantitation of Her2 receptor numbers on several breast cancer cell lines, including MCF7, BT-474, and MDA-MB-231, by using fluorescence-activated cell sorting analysis [17]. In that report, the absolute numbers of receptors per cell are 6- to 13-fold lower compared with our data, but the results are proportionally similar; that is, BT-474 had the highest receptors per cell, whereas MDA-MB-231 had the lowest of these three cell lines. Therefore, we have identified a panel of cell lines with varying levels of Her2 expression on the cell surface to use in subsequent experiments to assess the efficacy, specificity, and sensitivity of anti-Her2 antibody-conjugated magnetic nanoparticles.

### Binding of antibody-conjugated nanoparticles to Her2-expressing cell lines demonstrated specific binding based on Her2 expression

**Figure 3:**
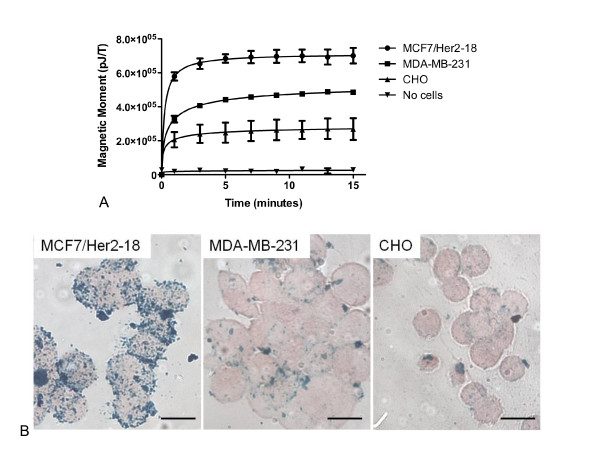
**SQUID detection of nanoparticle binding versus time**. **(a) **Cells (7.5 × 10^6^) from each breast cancer cell line or media alone were incubated with 0.8 mg of nanoparticles for 15 minutes on ice. SQUID measurements were taken every 2 minutes. The curves represent non-linear fits of the data. Data represent results from two separate experiments, and error bars represent the standard deviation. **(b) **Photomicrographs show 40× imaging of Prussian blue histochemical staining for the presence of iron oxide nanoparticles on cells incubated with anti-Her2 antibody-conjugated nanoparticles. Scale bars = 20 μM. Her2, human epidermal growth factor-like receptor 2; SQUID, Superconducting Quantum Interference Device.

To evaluate ligand-specific nanoparticle binding behavior, cell lines with varied Her2 expression, including MCF7/Her2-18, MDA-MB-231, and CHO, were incubated with anti-Her2 antibody-tagged nanoparticles for 15 minutes. Cells were assessed for nanoparticle binding by relaxometry every 2 minutes, starting 1 minute after nanoparticle addition. Results showed a sharp increase in relaxometry signal between the nanoparticles alone (time 0) and after 1 minute of incubation (Figure 3a). Signal was maximal after 5 minutes of incubation. The relaxometry signal increased with increasing Her2 receptor number; background due to anti-Her2-conjugated nanoparticles in the absence of cells was very low. MCF7/Her2-18 cells demonstrated the highest maximal relaxometry signal (700,500 pJ/T), MDA-MB-231 had an intermediate maximal relaxometry signal (435,500 pJ/T), CHO had a low maximal relaxometry signal (268,000 pJ/T), and media alone demonstrated a minimal background signal (29,000 pJ/T). After the relaxometry measurement, cytospin slide preparations were stained with Prussian blue to non-quantitatively visualize iron oxide nanoparticles histochemically. Qualitatively, MCF7/Her2-18 samples showed the highest level of cell-associated anti-Her2 antibody-conjugated nanoparticles, MDA-MB-231 cells showed intermediate binding of targeted nanoparticles, and CHO cells showed a low level of nanoparticle binding (Figure 3b); this is in agreement with the calculated number of Her2 receptors.

### Antibody-conjugated nanoparticles incubated with Her2-expressing cells were localized to the cell surface

**Figure 4:**
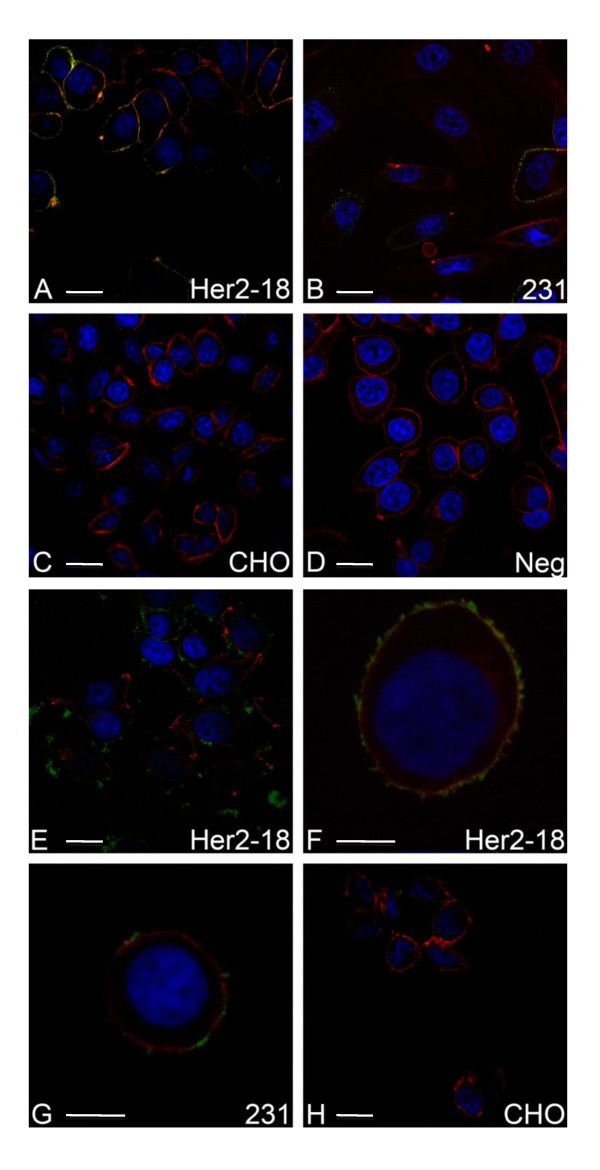
**Detection of cell-nanoparticle association by fluorescent immunodetection and confocal microscopy**. Her2 antigen was detected on the surface of MCF7/Her2-18 cells by indirect immunofluorescence assay **(a) **(green label), whereas MDA-MB-231 cells demonstrated little to no Her2 expression on the surface, although antigen was detected within cytoplasmic structures in some cells **(b) **(green label). CHO cells are negative for Her2 **(c)**, whereas negative control samples **(d) **demonstrate undetectable background labeling with the Alexa-fluor-labeled secondary antibody. Cells were counterstained with rhodamine phalloidin (red label) to visualize cell perimeters. A second set of cells was incubated with anti-Her2-labeled nanoparticles, and then fixed cells were incubated with fluorescently labeled secondary antibody to detect the anti-Her2-labeled nanoparticles and counterstained with rhodamine phalloidin (red label). Anti-Her2-conjugated nanoparticles were detected uniformly on the cell surface of MCF7/Her2-18 cells **(e, f) **(arrows). MDA-MB-231 cells demonstrate a low level of cell surface-associated anti-Her2-conjugated nanoparticles **(g) **(arrows), whereas no anti-Her2-conjugated nanoparticles were detected on CHO cells **(h)**. Scale bars = 10 μM (a-e, h) or 5 μM (f, g). CHO, Chinese hamster ovary; Her2, human epidermal growth factor-like receptor 2.

The above results suggest ligand-specific binding of antibody-conjugated nanoparticles to cells, so we next asked whether nanoparticles remain associated with the cell surface or whether they are internalized and, if so, what the time course of the internalization is. To address this, we incubated cells with antibody-conjugated nanoparticles as described and then fixed and detected the bound nanoparticles by indirect immunofluorescence by using Alexa 488-conjugated anti-mouse IgG. Cells were also incubated with rhodamine phalloidin to fluorescently label the actin cytoskeleton and were counterstained with DAPI or Topro-3 to visualize nuclei. In parallel assays, Her2 antigen was detected by using standard immunofluorescence microscopy. As shown in Figure 4, we detected abundant Her2 on the surface of MCF7/Her2-18 cells transfected with Her2 (Figure 4a). MDA-MB-231 cells appear to express Her2 in cytoplasmic vesicles but do not express the antigen on the cell surface, and this in agreement with previous observations (Figure 4b). CHO cells do not express any immunodetectable Her2 (Figure 4c). Using confocal microscopy and fluorescent detection of nanoparticles, we detected significant nanoparticle association with MCF7/Her2-18 cells (Figure 4e, f), reduced nanoparticle association with MDA-MB-231 cells (Figure 4g), and none with CHO cells (Figure 4h). These results are consistent with the quantitation of surface-expressed Her2 antigens (Figure 2), and with the detection of Her2 antigen by immunofluorescence (Figure 4a-c). Therefore, the binding of anti-Her2-labeled magnetic nanoparticles to live cells and their subsequent detection correspond directly to the localization and density of Her2 on cell surfaces. These results suggest that antibody-labeled nanoparticles represent a sensitive and accurate tool for detection of cells displaying surface-localized antigens.

### The SQUID relaxometry system detected antibody-conjugated nanoparticles bound specifically to breast tumor explants grown as xenografts

**Figure 5:**
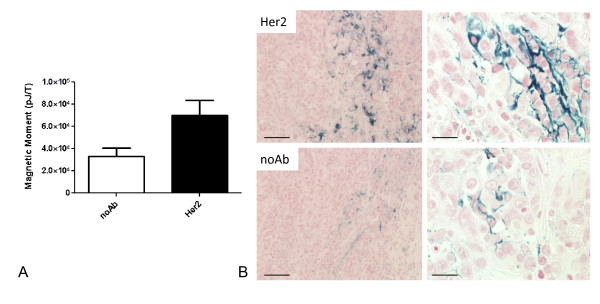
**Binding of nanoparticles to MCF7/Her2-18 tumors grown in mice**. **(a) **Excised tumors were injected with anti-Her2 antibody-conjugated nanoparticles or unconjugated nanoparticles (no Ab) and examined by SQUID. Data represent the mean and standard error measurement of four experiments (*P *= 0.049). **(b) **Photomicrographs of injected tissue with anti-Her2 or no Ab nanoparticles at magnifications of 100× (left) and 400× (right). Scale bars = 100 μM (left) and 25 μM (right). Her2, human epidermal growth factor-like receptor 2; SQUID, Superconducting Quantum Interference Device.

Cells in culture allow greater access of the nanoparticles to the cell surface than is anticipated in a solid tumor. To demonstrate the ability of the antibody-labeled nanoparticles to bind to breast cancer cells grown as tumors and to assess the capability of the magnetic relaxometry system to detect bound nanoparticles, we assessed binding of anti-Her2 antibody-conjugated nanoparticles to explanted MCF7/Her2-18 cell tumors grown as subcutaneous xenografts. Slices of MCF7/Her2-18 tumors were injected with nanoparticles conjugated to anti-Her2 antibody or unconjugated nanoparticles and washed to remove unbound nanoparticles. This experiment was repeated with four separate tumors and showed a significant (*P *= 0.049) increase in the magnetic relaxometry signal when anti-Her2 antibody-conjugated nanoparticles were injected compared with unconjugated nanoparticles (Figure 5a). To confirm that the difference in magnetic relaxometry signal was due to nanoparticle binding to tumor cells, paraffin-embedded tumor sections were stained with Prussian blue to detect iron nanoparticles within tumors (Figure 5b). A low level of Prussian blue staining is seen in tumors injected with unconjugated nanoparticles, and this likely reflects a degree of tissue trapping of unbound nanoparticles and is in agreement with the magnetic relaxometry signal detection in that sample. Importantly, increased Prussian blue staining is visible in the tumor slices microinjected with anti-Her2 antibody-conjugated nanoparticles compared with the unconjugated nanoparticles. Examining the anti-Her2 antibody-conjugated nanoparticle binding at higher magnification suggests that, as expected, the binding is cell surface-associated (Figure 5b). However, the distribution of the nanoparticles was not uniform, suggesting heterogeneity in antibody-conjugated nanoparticle access to tumor cells within the tumor environment. These results demonstrate that targeted nanoparticles produce an increased relaxometry signal as expected but that the distribution of targeted nanoparticles in tumors *in vivo *is non-uniform.

### The SQUID relaxometry method detected and localized nanoparticle-labeled breast cancer cells embedded within a breast phantom

**Figure 6:**
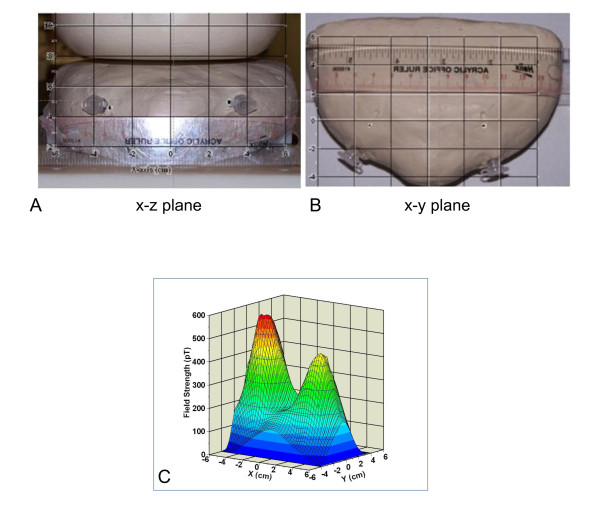
**Photographs of the breast clay phantom containing two vials of MCF7/HER2-18 cells**. **(a) **The x-z plane with z coordinate in the vertical direction. **(b) **The x-y plane with the × coordinate along the axis of the ruler. The small black dots (indicated by red arrows) are the 65% confidence limits for determining the positions of the centroid of the cells in the embedded vials. The size of the dots represents the uncertainty in the computed position of the sources. **(c) **A three-dimensional contour of the emitted magnetic relaxometry fields from the two sources contained in the phantom.

An important question regarding detection of tumors in patients is sensitivity. Sensitivity is affected by several parameters, including the sensitivity of the instrumentation, the intensity of the signal (influenced by intrinsic mechanisms and by the quantity of the labeled particles that can be achieved in the tumor), and the distance between the sensor and the tumor. Here, we demonstrate robust and spatially accurate detection of two sources each containing 3.75 × 10^6 ^nanoparticle-labeled cells embedded in a clay breast phantom, which accurately replicates a breast phantom routinely used in mammography imaging. Since both the human body and clay are transparent to low-frequency magnetic fields, the clay phantom closely simulates typical breast geometry and magnetic behavior. Figure 6a (x-z plane) shows the breast phantom directly below the sensor system, containing two vials of MCF7/Her2-18 cells labeled with Ocean SAO5 anti-Her2 antibody-conjugated nanoparticles. Both vials are approximately 2 cm in length and angled toward the center, and the cells are a spatially distributed source near the distal end. Figure 6b is a photo of the phantom taken from above and shows the x-y plane. The two indentations in the phantom in this view show the approximate ends of the vials within the phantom. The ruler placed on the phantom shows that the ends of the vials are approximately 6 cm apart. The sample stage was moved to 10 different locations within a 5-cm grid, giving a total of 70 measured field amplitudes. At each stage position, the magnetic fields were recorded and the resulting data were fit as described in Materials and methods. Only the two-dipole model produced reasonable fits since models assuming three or more sources did not yield any additional sources whose magnitude exceeded the measurement uncertainty. Figure 6c shows a three-dimensional contour plot of the magnetic fields from the phantom. The two large peaks correspond to fields from the two cell sources embedded in the phantom.

Table 1 gives the coordinates and magnitudes of the sources in the phantom (assuming 65% confidence limits). The two sources, each containing approximately 3.75 × 10^6 ^cells, produce the same magnetic moment within the errors at distances of 4.96 and 4.58 cm, respectively, from the sensor array (Figure 6b). The source positions calculated using the magnetic dipole method, shown as black dots in Figure 6, predict a separation of 6.23 cm, which is consistent with the distance between the two sources determined by a ruler. The uncertainties (65% confidence) in the position of these sources, as shown in Table 1, were on the order of 0.5 mm in the x-y plane (parallel to the sensor array) and 1.3 mm in the z direction. These results are shown graphically, appearing as black dots (whose size represents the confidence interval) in the x-z plane shown in Figure 6a. The confidence ellipses in Figure 6b, again appearing as black dots, fall within the vials and near the centroid of the cell samples as expected.

**Table 1:** Coordinates and magnitudes of sources in the breast phantom (assuming 65% confidence limits)

	X, cm	Y, cm	Z, cm	Magnetic moment, pJ/T
Source 1	3.05	-0.36	4.96	3.90 × 10^5^
Uncertainty	0.06	0.05	0.14	3.21 × 10^4^
Source 2	-3.17	-0.33	4.58	3.92 × 10^5^
Uncertainty	0.04	0.04	0.12	2.95 × 10^4^

These results indicate the accuracy of this method for locating nanoparticle-labeled cells in an actual human breast, given a similar number of measurement points, acquired by moving the subject or the use of a larger sensor array. They also illustrate that the positions of the sources within the phantom are obtainable in three dimensions and that the intervening clay medium has no influence on this determination. Several biomagnetic systems that use 37 channels have been developed for magnetoencephalography [7]; these systems can be readily adapted to the magnetic relaxometry application, and the data set used above simulates their usage.

### The SQUID relaxometry system detected 1 million cells at a depth of 4.5 cm

**Figure 7:**
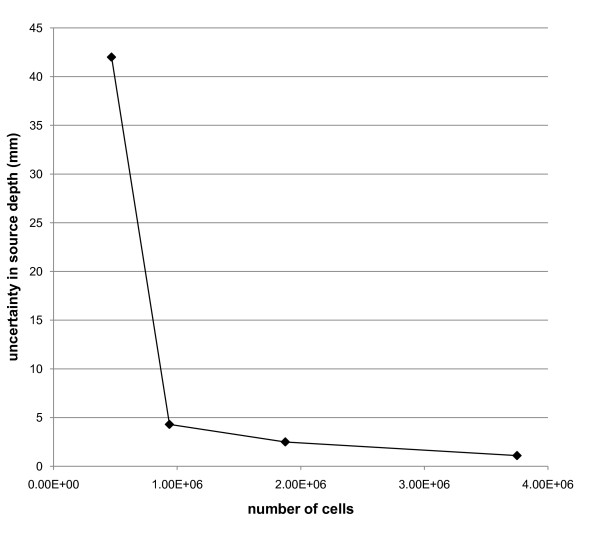
**Uncertainty in the detected source depth versus cell number for samples containing magnetically labeled MCF7/Her2-18 human breast cancer cells located 4.5 cm below the SQUID sensors**. The data demonstrate a detection sensitivity, obtained using our current hardware and SHP-30 SAO5 nanoparticles, of 940,000 cells at a depth of 4.5 cm. Theoretically, further improvements in the hardware and nanoparticles will result in even higher sensitivity. Her2, human epidermal growth factor-like receptor 2; SQUID, Superconducting Quantum Interference Device.

The phantom results indicate that two magnetically labeled cell samples (each containing 3.75 × 10^6 ^cells) can be reliably detected and located at a depth of approximately 5 cm by using our technique. To further explore our detection limits, one of the cell sources used in the phantom measurements was diluted serially to produce a set of samples with 3.75 × 10^6^, 1.9 × 10^6^, 9.4 × 10^5^, 4.7 × 10^5^, and 2.3 × 10^5 ^magnetically labeled MCF7/Her2-18 cells. Each cell sample was placed 4.5 cm below the sensor system, and SQUID measurements were obtained at five different sample stage positions to accurately determine the spatial coordinates and magnetic moment of each cell sample. Figure 7 shows the resulting uncertainty (95% confidence; that is, two standard deviations) in the fitted value of the source depth as a function of cell number. These data demonstrate a detection threshold of about 10^6 ^cells, below which the uncertainty in the source location increases precipitously. At the threshold, the uncertainty in the detected source depth was approximately 4 mm, and the uncertainty in the magnetic moment (not shown) was roughly 20%. Above the threshold, a gradual decrease in uncertainty with increasing cell number is observed because of the increasing signal-to-noise ratio.

The thickness of a human breast, compressed for mammography, averages 4.4 cm (range of 1.4 to 7.2 cm) [18]. Thus, in the compressed geometry, the maximum depth of a tumor would fall between 0.7 and 3.6 cm (that is, half the thickness). Because the minimum distance between the SQUID sensor and any source will be approximately 1 cm (due to the insulation surrounding the superconducting sensors), characterizing the sensitivity of our detection system at depths in the range of 2 to 5 cm is particularly relevant when considering the detection of primary breast tumors in humans. We have demonstrated the ability to detect as few as 1 million optimally labeled breast cancer cells at a depth of 4.5 cm. For our sensor system, which uses second-order gradiometers for the pick-up coils, we have determined that the SQUID signal detected from a source is proportional to z^-3.4 ^for the range of z (depth) considered here. With this proportionality, our current detection limits at z = 2, 3, 4, and 5 cm are calculated to be 6.0 × 10^4^, 2.4 × 10^5^, 6.3 × 10^5^, and 1.3 × 10^6 ^cells, respectively, on the basis of the detection threshold of 940,000 cells observed at 4.5 cm. Theoretically, even higher detection sensitivity can be achieved [12]; anticipated improvements in both the hardware (for example, improved electromagnetic shielding) and the magnetic nanoparticles (for example, reduced size polydispersity) are expected to lead to a further increase of the detection sensitivity by at least two orders of magnitude. Our current detection sensitivity (1 million optimally labeled cells at 4.5 cm) is approximately 1,000 times fewer cells than the size at which a tumor is first palpable (~10^9 ^cells) and 100 times fewer cells than the detection limit for x-ray imaging (~10^8 ^cells) [19]. However, we note that the degree of labeling that will be achievable *in vivo *and the level of non-specific background that will be observed after systemic nanoparticle delivery remain to be determined. Whereas breast thickness would affect our detection sensitivity (because of the dependence of magnetic field amplitude on distance from the source), radiographic breast density would not affect our method, because all tissue is transparent to the very low-frequency magnetic fields employed in our method. Furthermore, our method would not increase cancer risk, because it avoids the use of ionizing radiation. The potential sensitivity of our system and the lack of ionizing radiation would also be advantages for monitoring tumor size during therapy.

## Conclusions

These results with tumor cell-specific antibody-bound magnetic nanoparticles demonstrate the feasibility of using targeted nanoparticles in combination with SQUID-detected magnetic relaxometry to detect breast tumor cells. First, the magnetic relaxometry method will be instrumental in the development of targeted nanoparticles because it provides a rapid and highly sensitive way to test the validity of nanoparticles targeted to surface antigens. Methods for testing nanoparticles are critical to the development of targeted nanoparticle cocktails for breast cancer detection as well as individualized targeted particles for monitoring treatment and metastases in breast cancer patients with a known histological tumor profile. Moreover, SQUID-detected magnetic relaxometry shows great potential for improving breast cancer detection sensitivity and specificity over current methodologies, which, when combined with advances in tumor-specific delivery of detection and therapeutic nanoparticles, will eventually contribute to improved clinical monitoring and outcomes for patients with breast cancer.

## Abbreviations

CHO: Chinese hamster ovary; DAPI: 4',6-diamidino-2-phenylindole; EDTA: ethylenediaminetetraacetic acid; FBS: fetal bovine serum; FITC: fluorescein isothiocyanate; Her2: human epidermal growth factor-like receptor 2; NGS: normal goat serum; PBS: phosphate-buffered saline; SQUID: Superconducting Quantum Interference Device; TEM: transmission electron microscopy.

## Competing interests

NLA has equity interests in ABQMR (Albuquerque, NM, USA) and nanoMR (Albuquerque, NM, USA); neither company sponsored this work. The other authors declare that they have no competing interests.

## Authors' contributions

HJH participated in experiment design, data collection and interpretation, and the writing of the manuscript and is a contributing author. KSB participated in experiment design, experiment execution (Prussian blue assays, SQUID measurements, and *in vivo *analysis), data collection and interpretation, and the writing of the manuscript. HJH and KSB contributed equally to the manuscript. NLA participated in experiment design, experiment execution (SQUID measurements and phantom experiments), data collection and interpretation, and the writing of the manuscript. DML participated in experiment design, experiment execution (site density measurements, SQUID measurements, and cell culture), data collection and interpretation, and the writing of the manuscript. RB participated in experiment execution (confocal microscopy) and data collection and interpretation. DF participated in experiment execution (TEM measurements) and data collection and interpretation. TCM participated in nanoparticle characterization and data collection. DLH participated in experiment design and nanoparticle characterization. JET participated in execution of preliminary *in vitro *experiments. TET participated in software development and data analysis and interpretation. HCB participated in analysis and interpretation of nanoparticle characterization data. RSL participated in experiment design and data interpretation. ERF participated in experiment design, breast phantom measurements, calculations of source locations, data interpretation, and the writing of the manuscript. All authors read and approved the final manuscript.
